#  Vozes indígenas na saúde: retomando territórios de fala na silenciosa noite colonial 

**DOI:** 10.1590/0102-311XPT080323

**Published:** 2023-07-17

**Authors:** Adriana Rosa Cruz Santos

**Affiliations:** 1 Instituto de Psicologia, Universidade Federal Fluminense, Niterói, Brasil.

O ano de 2023 começou com a explosão, na mídia, da emergência humanitária yanomami: crianças e adultos mortos de fome, malária, garimpo ilegal, racismo, silêncio. Há centenas de anos desconhecemos quem somos, de onde viemos e quem “sustenta o céu” que nos abriga ^1^. Alguns dos efeitos do empreendimento capitalista colonial ^2^ são a desarticulação às redes de pertencimento originárias, a desconexão com cosmopolíticas não antropocêntricas e a insularização individualizante.

Portanto, não é trivial estarmos aqui hoje discutindo sobre o reflorestamento contracolonial proposto por *Vozes Indígenas na Saúde: Trajetórias, Memórias e Protagonismos*^3^. O reflorestamento como caminho de enfrentamento da urgência climática planetária nos convoca a adotar outras sintaxes, outros vocabulários, outras políticas de subjetivação e a sintonizar outras vozes. No Capitaloceno, Ailton Krenak ^4^ (p. 65) convida a “...*provocar uma experiência de florestania começando por contestar essa ordem urbana sanitária*”, enquanto as mulheres indígenas guerreiras da ancestralidade nos chamam a reflorestarmentes.

“*É possível vivermos e convivermos de outra forma, com outras epistemes, a partir de cosmologias ancestrais. Cuidar da Mãe Terra é, no fundo, cuidar de nossos próprios corpos e espíritos. Corpo é terra, floresta é mente. Queremos reflorestar as mentes para que elas se somem para prover os cuidados tão necessários com nosso corpo-terra*” ^5^.

Florestania, corpo-terra, reflorestar mentes: noções que emergem de uma cosmopolítica complexa, em que a monocultura do pensamento colonial ^6^ dá lugar à multiplicidade ontológica e epistêmica dos povos originários. A primeira coisa que aprendemos no livro organizado por Ana Lucia de Moura Pontes, Vanessa Hacon, Luiz Eloy Terena e Ricardo Ventura Santos é que, na perspectiva indígena, não é possível pensar isoladamente a saúde. “Corpo é terra” e saúde é território, como adverte Lourenço Krikatí: “*Como é que nós teremos saúde se não temos território?*” ^3^ (p. 73).

O território, por sua vez, não se limita à questão fundiária (ainda que essa dimensão material seja fundamental), mas é uma teia complexa de relações que articula entes humanos e mais-que-humanos, o visível e o invisível, sustentando a vida em toda a sua profusão.

Esse e outros importantes operadores do pensamento indígena emergem no livro originado da pesquisa *Saúde dos Povos Indígenas no Brasil: Perspectivas Históricas, Socioculturais e Políticas*, desenvolvida na Escola Nacional de Saúde Pública Sergio Arouca, Fundação Oswaldo Cruz (ENSP/Fiocruz). A obra reúne relatos de indígenas de diferentes povos sobre os processos de luta e construção de uma política de saúde indígena no âmbito do Sistema Único de Saúde (SUS). É preciso dizer em alto e bom som os seus nomes: Ailton Krenak, Zezinho Kaxarari, Chico Apurinã, Letícia Yawanawá, Carmem Pankararu, Lourenço Krikati, Ivani Gomes Pankararu, Davi Kopenawa Yanomami, Álvaro Tukano, Iolanda Pereira Macuxi, Clóvis Ambrosio Wapichana, Jacir de Souza Macuxi e Megaron Txucarramãe Mebêngôkre.

Trata-se, portanto, de um importante documento histórico que registra vozes tradicionalmente silenciadas de indígenas protagonistas na construção de políticas de saúde para essa população, que guarda, entre outros, os desafios - também históricos - de superação das marcas e violências da colonialidade. Como afirma Célia Xakriabá: “*não basta reconhecer os conhecimentos indígenas, é urgente reconhecer os conhecedores. Porque nós emergimos de uma ciência que nasce do território e carregamos essa ciência dentro de nós*” ^3^ (p. 324). Reconhecer os conhecedores, restituir-lhes a autoria da trajetória e do conhecimento produzido e tornar visível sua contribuição ao movimento de luta pela saúde são gestos simultaneamente epistemológicos e políticos, que ativamente rompem com o silenciamento colonial.

O percurso vivido e apresentado pelas lideranças indígenas não é simples: como conciliar a noção ampliada de saúde dos povos indígenas, que inclui as dimensões coletiva e espiritual do viver (em que a terra, os rios e outros entes mais-que-humanos fazem parte do “diagnóstico” e do “tratamento”), com as práticas individualizadas de cuidado do modelo biomédico branco? Como construir um sistema de saúde que utilize as tecnologias biomédicas de cuidado sem negligenciar os saberes ancestrais de parteiras, rezadeiras, pajés e os saberes compartilhados coletivamente sobre a potência curativa dos vegetais e dos rituais, que permitiu aos povos indígenas viver em harmonia com o que se convencionou chamar “natureza” por milhares de anos, até a invasão europeia? Como reivindicar ao Estado brasileiro o direito à saúde, sem subsumir na categoria homogeneizante e etnocida de cidadão brasileiro?

Esses desafios atravessam décadas de luta e engajamento de diferentes organizações indígenas e de sua relação com entes estatais - como a Fundação Nacional dos Povos Indígenas (Funai) e a extinta Fundação Nacional de Saúde - e organizações não governamentais. Nesse processo, os indígenas propuseram diferentes estratégias de organização e gestão, exerceram ativamente o controle social, participaram da implantação dos Distritos Sanitários Especiais Indígenas (DSEI), da elaboração da Política Nacional de Atenção à Saúde dos Povos Indígenas (PNASPI), em 2002, e, finalmente, da criação da Secretaria Especial de Saúde Indígena (SESAI), no âmbito do Ministério da Saúde, espaço de maior envergadura e reconhecimento institucional em um processo incansável.

Ainda que estejamos muito distantes de um processo de reparação histórica do Estado brasileiro - tal como supôs o entendimento do Supremo Tribunal Federal (STF) ao reconhecer o direito ao território indígena Raposa Serra do Sol, referido por Luiz Eloy Terena, na página 344 ^3^ -, a criação do Ministério dos Povos Indígenas em 2023 e a majoritária participação de indígenas na gestão e composição das equipes do recém-criado ministério e da Funai fazem ressoar as palavras de Carmem Pankararu: “*nós queremos que o Estado reconheça que estamos em um país miscigenado, multicultural, multiétnico e que precisamos desse entendimento e respeito nas estruturas de saúde*” ^3^ (p. 161).

Esse Brasil multiétnico, capaz de múltiplas formas de cultura e expressão, aparece nos traços do artista wapichana Gustavo Caboco, os quais compõem as ilustrações que dançam entre os relatos, conferindo vida e movimento à obra, num gesto de recriação da memória, que atravessa o livro como uma flecha, encantando as palavras. A interlocução que encerra o livro acontece entre as lideranças indígenas Célia Xakriabá (eleita recentemente Deputada Federal) e Luiz Eloy Terena, assessor jurídico da Articulação dos Povos Indígenas do Brasil (APIB). O epílogo destaca importantes inflexões presentes nos relatos, ampliando a relevância desse documento histórico com a análise e a perspectiva de quem “herda a luta” e recebe dos ancestrais encantados a direção e a força.

Por fim, cabe ressaltar a importância desse livro para todes aqueles que se interessam, trabalham ou lutam pela/na saúde coletiva e pela retomada indígena de seu espaço sagrado, físico e imaterial. Ou melhor: para todes aqueles, além das fronteiras raciais impostas pela colonialidade, tocados pela experiência de liminaridade que a crise climática global provocada pelo capitalismo predatório vem produzindo. As dissonâncias que emergem no encontro entre o modo de vida indígena e a saúde do branco deflagram problematizações em torno das noções de saúde-doença, de tecnologias de cuidado, de corpo, de indivíduo, do que é (e o que pode ser) viver. Ailton Krenak afirma: “...*nossa maneira de cuidar baseada em um tipo de pensamento sobre o corpo, sobre o ser, que opera em conjunto com outras entidades: soprando fumaça, achando os pontos, olhando, vendo o que está alinhado, o que está desalinhado. Há uma sensibilidade para ver quando o espírito e o corpo da pessoa estão em harmonia e quando estão dissociados. Só quando esses dois, quando esses múltiplos - porque não são só dois - atributos do ser experimentam alguma desconexão, é que entra aquele evento que chamam de doença. E que não é uma coisa só, não tem um diagnóstico*^3^ (p. 55).

Múltiplos são os atributos do ser e as dimensões do viver. Deixemos que o ar que inspiramos - ente mais-que-humano que entra e sai sem cessar dos nossos corpos - sopre, abra espaços e crie caminhos de cura e reconexão com a Terra, esse plano/planeta múltiplo que nos abriga e habita. Realinhamentos entre corpo e espírito, entre o que somos e o que podemos ser. Reflorestemos nossas práticas de cuidado e retomemos, em nós, amplitudes de viver. O dia está quase nascendo.
